# Community-Engaged Development of Equitable and Scalable Mobile Health Tools for Tobacco Treatment: The Healthy Lungs Trial Experience

**DOI:** 10.1016/j.chpulm.2024.100127

**Published:** 2025-01-24

**Authors:** Joanna L. Hart, Tamar Klaiman, Michael Scott, George M. Fernandez, Dorothy Sheu, Aerielle Belk, Jasmine A. Silvestri, Jannie Kim, Scott D. Halpern, Nsenga Farrell

**Affiliations:** a Palliative and Advanced Illness Research Center, University of Pennsylvania, Philadelphia, PA; b Division of Pulmonary, Allergy, and Critical Care, Department of Medicine, University of Pennsylvania, Philadelphia, PA; c Department of Medical Ethics and Health Policy, University of Pennsylvania, Philadelphia, PA; d Michael J. Crescenz VA Medical Center, Philadelphia, PA; e Center for Black Health and Equity, Durham, NC; f Latino Connection, Color and Culture, Harrisburg, PA; g CHDI Foundation, New York, NY

**Keywords:** community engagement, health equity, patient-centered design, tobacco use disorder

## Abstract

**Background:**

Tobacco use has a disproportionate impact on older, medically underserved adults. Mobile health (mHealth) tools hold promise for increasing reach of treatment options, yet introduce new barriers to access and use.

**Research Question:**

How can investigators incorporate patient and community input into the design and testing of accessible, scalable, and equity-promoting mHealth tobacco treatment tools?

**Study Design and Methods:**

We present a model for mHealth tobacco treatment tool development using a longitudinal community-partnered design process. We iteratively developed and refined tools used in a large, pragmatic trial. First, a stakeholder advisory committee (SAC) convened with members including individual patients and representatives from patient and health equity advocacy groups, community and government public health services, clinical program leads, and health system and insurance leaders. Second, we conducted a patient needs assessment to confirm or expand on SAC recommendations using semistructured interviews among patients meeting ≥ 1 medically underserved criteria who smoked tobacco daily. Transcribed interviews were coded and analyzed for patterns of patients’ desired design elements.

**Results:**

The SAC recommended key strategies to promote cultural relevance of the tools, maximize engagement of participants, and prevent attrition, which were incorporated into the intervention and trial design. To further refine the approach, we completed interviews with 39 patients from November 2020 to September 2021. Many respondents used telemedicine tools with their clinicians yet were skeptical of their use for tobacco treatment due to lack of facility with mobile technologies. Patients recommended direct support options, avoidance of novel smartphone applications, and customizable features.

**Interpretation:**

We provide a model for patient-centered design that incorporates community engagement through longitudinal advisors and wider representation of patients. Longitudinal community engagement that incorporates broad patient perspectives facilitates effective development and deployment of mHealth tools to maximize responsiveness to patient and community needs.

**Take-Home Points**

**Study Question:** How can patient and community input be incorporated into the design and testing of health tools, especially those intended to be both scalable and equitable?

**Results:** Tools should incorporate features that mimic typical use by patients and provide opportunities for direct support, customization, and a personal touch to promote patients feeling valued.

**Interpretation:** Community-engaged codesign of health tools may include both longitudinal community engagement and broader patient representation to incorporate broad patient perspectives while providing opportunities to iterate on key tool elements.

Tobacco use has a disproportionate impact on older, medically underserved adults.^1^, ^2^, ^3^ Tobacco treatment, including pharmacologic and behavioral tools, may mitigate long-term health disparities.^4^ However, many affected individuals lack sufficient access to tobacco treatment, particularly those living in rural areas and those with low socioeconomic status.^5^ Mobile health (mHealth) tools hold particular promise for reaching these groups. In many ways, mHealth support is better designed to respond to individuals’ in-the-moment nicotine withdrawal symptoms, social triggers, or tobacco cravings than traditional intermittent in-person approaches.^6^^,^^7^ However, little is known regarding how to design these tools to overcome barriers among the groups who carry the largest burden of tobacco-related disease in the United States. Without sufficient tailoring, mHealth tools may widen health disparities by preferentially expanding access in groups who already have more resources.^7^

We developed mHealth tobacco treatment tools responsive to the needs of diverse groups of patients as part of a trial of tobacco treatment strategies that could be widely implemented by lung cancer screening programs.^8^ The Healthy Lungs trial is a large, highly pragmatic randomized trial at 4 US health systems that compares the effectiveness of 4 interventions to promote sustained abstinence from tobacco smoking at the time of lung cancer screening referral.^8^ To promote pragmatism, the trial relies on mHealth tools to enroll participants and deliver tobacco treatment interventions. The Healthy Lungs trial specifically seeks to reach individuals using tobacco and self-identifying as meeting ≥ 1 demographic characteristics: household income < 200% of the federal poverty line; less than a high school level of formal educational attainment; Black or African American, Hispanic, or Latinx race or ethnicity; and/or self-identifying as living in a rural area.

We conducted a community-engaged codesign process to help design these tools for maximal feasibility, uptake, and sustained use among a diverse group of older individuals. This iterative process developed and refined tobacco treatment tools including their content, delivery, and key elements before trial launch. Codesign can improve user-centeredness, implementation, and trust, particularly among groups facing challenges to uptake of mHealth,^9^, ^10^, ^11^, ^12^, ^13^, ^14^, ^15^, ^16^, ^17^ but often fails in execution or is overlooked in mHealth designs.^10^^,^^18^ This paper describes a general, collaborative model of a longitudinal community-partnered design process and identifies specific design features important for equitable mHealth tobacco treatment tools.

## Study Design and Methods

### Stakeholder Advisory Committee

**Figure 1:**
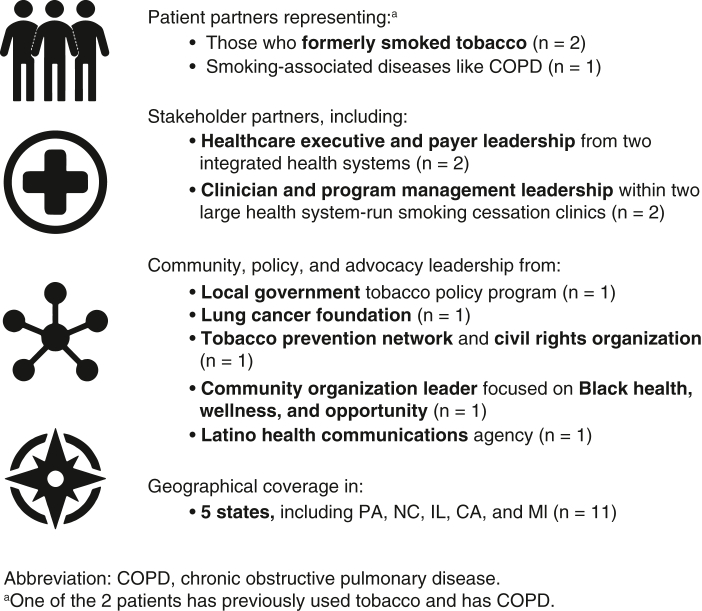
Stakeholder advisory committee members.

We convened our stakeholder advisory committee (SAC) in 2018. We recruited SAC members after identifying relevant stakeholder groups and representative organizations, identifying shared missions and values, sharing our individual and collective strengths and challenges, and probing current barriers to achieving desired health outcomes. We identified members through existing professional networks, past collaborations or research participation, and direct outreach after searching for aligned organizations. Our SAC consisted of 11 members (Fig 1) including patients and organizational representatives. We specifically sought to represent patient and organizational perspectives that acknowledge and represent the intersectionality of the target demographic groups in the Healthy Lungs trial. During the initial phase, we shared goals and expectations for the research and participation in the committee. Stakeholders and the investigative team met in June 2019 and agreed on a charter governing the group including voluntary participation agreements. SAC members were offered $50 per hour for their work. This initial meeting included a colearning session on patient-centric and stakeholder-engaged research practices.

The SAC members codesigned the trial with investigators before launch, including by refining key aspects of study structure, design, and implementation. We identified design elements that required stakeholder input through investigative team meetings, regular contact with SAC lead (M. S.), and SAC meetings. Based on feedback and stakeholder goals, we established 4 working groups to further ensure the research design would meet patient participants’ needs and those of the clinical sites at which they seek care: (1) resources and technology used by patients, (2) adaptation of a specific tool (episodic future thinking [EFT]) tested in the trial, (3) patient-facing materials, and (4) health system integration. SAC members self-selected working group(s) they thought they could contribute to most and that were most aligned with their goals for participation. These working groups provided synchronous (virtual) and asynchronous feedback and cocreation of trial materials. SAC members provided feedback via evaluative surveys on their contributions and engagement processes.

### Semistructured Interviews

To provide greater patient perspective, we conducted a qualitative study using semistructured interviews among patients meeting ≥ 1 medically underserved criteria (ie, consistent with Healthy Lungs trial demographic eligibility criteria and subsequently defined) who smoked tobacco daily. We developed and operationalized the study alongside SAC members, including partnering with an affiliated community health organization to recruit participants. At a September 2020 SAC meeting, we refined the interview guide with goals of accessibility, tailoring to patients, and informative for mHealth tool development (e-Appendix 1). We also consulted with selected primary care leaders in 2 virtual meetings to adapt and implement the recruitment of patients from clinics serving rural populations (ie, patients with Rural-Urban Commuting Area Codes ≥ 2^19^). The Institutional Review Board of the University of Pennsylvania approved all procedures (#843833).

Interviewers were research staff trained in interviewing skills, including probing and reflective listening, and were women of diverse racial and ethnic backgrounds. All interview questions were open-ended. Interviewers used standard prompts to probe specific themes after participants offered spontaneous insights to each question. Subsequent questions followed from participants' responses, with the interviewer pursuing themes as they arose and seeking clarification or elaboration when appropriate. A fluent Spanish-speaking research staff member (J. K.) translated the interview guide into Spanish and conducted Spanish language interviews.

### Sampling Strategy and Participants

For these semistructured interviews, potentially eligible outpatients were identified through the electronic health record (EHR) of a single large health system. Recruitment occurred before launch of the trial. We randomly selected outpatients who were at least 50 years of age and had EHR-documented daily tobacco use. We sent batches of mailed recruitment letters asking patients to complete an online screening survey to determine eligibility. We purposefully sampled across key demographics available in the EHR (eg, Rural-Urban Commuting Area code ≥ 2 based on ZIP code^19^) to ensure diverse representation of perspectives. The screening survey included demographic questions and confirmation of tobacco use behavior to determine study eligibility. Participating patients completed a demographic questionnaire reporting their smoking status, age, race, gender, ethnicity, income relative to household size, and education. Patients were eligible to participate in interviews if they were using tobacco daily, were 50 to 80 years of age (based on existing lung cancer screening guidelines^20^), and represented one of the target demographic groups (eg, self-reported household income < 200% of the federal poverty line; less than a high school level of formal educational attainment; Black, Hispanic, or Latinx race or ethnicity; and/or living in a rural area). Participants were required to have conversational fluency in English or Spanish. We continued to recruit patients until we had reached thematic saturation and sufficient information power based on concurrent analysis.^21^ To achieve diversity, we reviewed potential participants’ self-reported demographics and selectively invited them for participation.

Patient participants were also recruited through 2 supplemental sampling strategies to improve diversity and inclusion of patients not affiliated with the health system. First, we recruited in partnership with Latino Connection. Latino Connection develops outreach and education targeted at reaching and empowering low-income, underserved Latino communities to improve health. Trained [redacted] staff, as part of their state-supported COVID-19 mobile response unit outreach to low-income urban and rural areas in Pennsylvania, recruited and assessed for eligibility individuals engaging in their outreach sessions. Second, we conducted snowball sampling, in which participating patients provided information about the study to personal contacts who were then assessed for eligibility using the same approach.

Eligible and willing patients were contacted by research staff via phone. All participants provided verbal informed consent before the audio-recorded interview and received $50 for their time. Interviews were transcribed and translated (if needed) using a professional transcription service that removed identifying information.

### Analytical and Statistical Approaches

Project staff reviewed all transcripts for quality assurance to address missing data and ensure deidentification. We then entered all transcripts into NVIVO 12.0 (QSR International) to facilitate analysis. Investigators developed a preliminary codebook inductively based on emerging themes and deductively using prior literature review of key mHealth considerations and tobacco treatment barriers. All coding was overseen by a PhD-trained qualitative researcher (T. K.). Research staff (A. B., J. K., J. S., S. S.) trained in qualitative methods conducted the coding. The codebook was iteratively revised as additional transcripts were reviewed. Through asynchronous working groups with the SAC, we codrafted the codebooks. We distributed draft codebooks with key interview passages as corresponding examples. The SAC provided feedback on alignment of the passages with codes and participated in further adapting the interview guide and codebook.

Once a near-final version of the codebook was agreed on (e-Appendix 2), research staff coded all transcripts, with at least 20% coded by multiple staff members to assess reliability and prevent coding drift. Research staff met at least weekly during coding to resolve uncertainties, reaching consensus on any disagreements, iteratively revising the codebook, and re-reviewing previously coded transcripts as necessary. We conducted interviews and concurrent data analysis until we reached thematic saturation and sufficient information power (ie, had sufficiently represented each of our target demographic groups) based on continuous review of the data. During standing meetings, we discussed progress toward demographic representation and emerging themes. We then analyzed the coded content and developed thematic memos describing patterns with attention to themes within and across demographic groups. Once new interviews yielded no additional insights, we stopped recruitment, having reached saturation.^21^, ^22^, ^23^

In December 2021, we held a virtual SAC summit that included a workshop focused on refining the interpretation and presentation of the interview study findings. We specifically discussed ways to practically apply study findings within the main Healthy Lungs trial. Analysis of participant engagement with resulting tools, and their effectiveness in achieving smoking abstinence, will be presented elsewhere.

## Results

### SAC Contributions

#### SAC Influence on Key Study Components

On evaluative surveys, SAC members reported contributing most to ensuring the trial accounted for patients’ needs and preferences, addressing real-word barriers to carrying out the trial, and adapting the trial to patients’ sociocultural backgrounds. Members contributed to naming the trial and selecting a logo and color scheme that would appeal to potential participants, reducing survey burden at the time of enrollment while prioritizing collection of key data, establishing the frequency and content of communication and task reminders sent to trial participants, selecting trial participant compensation amounts, and helping to adapt evidence-based tools to fit patient and pragmatic needs.

#### Strategies to Support Sustained Participant Engagement

SAC members recommended several strategies that we implemented to maximize engagement of trial participants and prevent attrition. First, specific to the pragmatic trial component of the tobacco tools, the SAC recommended opt-out consent with presentation of the low-risk trial of efficacious interventions as a program available to participants, rather than as part of a study. Together with the SAC, we obtained approval for this approach from the institutional review board overseeing the trial.^8^ Second, they encouraged delivery of all Healthy Lungs trial mHealth tools via text messages given the target population’s more variable access to smartphone technology and high-speed internet. Third, we adopted the SAC recommendation to provide a hotline for personalized support via telephone in English or Spanish. Fourth, we sent all Healthy Lungs participants a welcome packet by mail based on SAC feedback. Although the tobacco treatment mHealth tools were delivered electronically, a paper packet with branded materials codeveloped with the SAC provided participants with technical and content support materials to improve their ability to access and understand the mHealth tools. The intention was for the target population of older adults to feel supported in their use of the mHealth tools, provide low-tech access to key materials and content, and improve accessibility of and engagement with technology-dependent resources.

#### Example of Tool and Resource Codevelopment

Finally, the SAC collaboratively adapted the EFT tool and codeveloped 2 animated videos (Kindea Labs) introducing the tool to participants. EFT tools typically require hours of staff time on education, personalization, and coaching of each participant, thereby limiting their feasibility and scalability.^24^, ^25^, ^26^, ^27^ Aided by the SAC and EFT experts, we identified the critical elements necessary for EFT fidelity and uptake. The SAC and investigative team then developed an adapted EFT tool that could be pragmatically delivered via website personalization and subsequent text message delivery, which will be detailed separately. The SAC codeveloped culturally appropriate and relevant content and prompts for tool personalization. We then story-boarded 2 animated videos, drafted scripts, and iteratively revised video drafts to provide 2 videos that provided detailed, culturally relevant content to (1) introduce EFT to participants and (2) advise them on effective use of the tool. These videos were available in English and Spanish, including subtitles for greater accessibility. The narrator was an SAC member.

### Semistructured Interviews

We recruited and interviewed participants from November 2020 to September 2021. We mailed outreach letters to 236 health system patients and 60 completed the eligibility survey for a response rate of 25.4%. Through our community outreach, 25 additional individuals completed eligibility surveys. In total, we identified 68 patients eligible to participate. We offered interviews on this topic to 39 patients, all of whom participated. Other eligible respondents participated in interviews on other topics.^28^ Four interviews were conducted in Spanish. Participants included 16 individuals (41.0%) identifying as Black or African American, 4 (10.3%) identifying as Latinx or Hispanic, and 20 (51.3%) identifying as women (Table 1). All data coded by > 1 coder had a kappa statistic > 0.8, indicating high reliability in code applications across coders and during the data analysis period. We identified 3 main themes.

**Table 1:** Characteristics of Interview Participants (N = 39)

Characteristic	No. (%)
Age, y	
50-54	3 (7.7)
55-59	10 (25.6)
60-64	14 (35.9)
65-69	10 (25.6)
70-74	2 (5.1)
≥ 75	0 (0.0)
Gender	
Man	19 (48.7)
Woman	20 (51.3)
Race	
White or Caucasian	19 (48.7)
Black or African American	16 (41.0)
Asian or Asian American	0 (0.0)
Native American	0 (0.0)
Other	4 (10.3)
Ethnicity	
Hispanic or Latino	4 (10.3)
Not Hispanic or Latino	35 (89.7)
Highest level of formal educational attainment	
Did not graduate from high school	7 (17.9)
High school degree or equivalent (eg, GED)	19 (48.7)
Some college but no degree	4 (10.3)
Associate’s or bachelor’s degree	5 (12.8)
Graduate degree	4 (10.3)
Income category based on household size	
< 200% FPL	24 (61.5)
≥ 200% FPL	12 (30.8)
Not reported	3 (7.7)
Self-reported rurality	
Lives in a rural area	4 (10.3)
Does not live in a rural area	35 (89.7)

FPL = federal poverty level; GED = General Educational Development Test.

#### Increasing Comfort With Telemedicine

Many respondents used telemedicine tools with their clinicians, particularly during the COVID-19 pandemic, and found them relatively easy to use with support (Table 2). Some patients had technologic challenges due to personal facility with technology or access to high-speed internet. Many were skeptical of telemedicine or other mHealth at first but became more comfortable with their use over time. Although some appreciated the convenience of not having to travel to appointments when using mHealth tools in place of face-to-face appointments, most respondents preferred in-person appointments.

**Table 2:** Patient Perspectives on mHealth Tool Use

Theme	Quotes
Increasing comfort with telemedicine	“Sometimes it could be emotionally one of those days when you feel like not going outside, which are things that happen every so often. I think that videocall thing is excellent, as I told my therapist, this is something they should keep doing after the pandemic, in cases like that where you don’t feel good, you see, just through videocall, so that neither the doctor loses that appointment nor the patient misses out.” (Patient 010) “I’m a lot more comfortable [with telemedicine] right now. I wasn’t, it took me a while to get comfortable with them. It probably took me since the beginning of the [COVID-19] pandemic and when I’m finally like adding these with different things that I’m doing.” (Patient 159)
Anticipated challenges in using mHealth tools	“I’m not too good with smart phones, because I don’t have one. Computers I’m like no good with . . . I know how to turn them on, but like, I had 1 year ago, they’re having a problem, I guess, with the Wi-Fi. So, I’m having problems even getting on the damn Internet. So, I’m going to try to use my girlfriend’s computer. She uses that more. So I’m very uncomfortable with smart phones and the computers.” (Patient 154) “I use [my smartphone] for the Internet, I pay bills, get texts an . . . email, Internet, pay bills.” (Patient 132) “Oh, well my sons are pretty good. Like if I have a question about [phones]—they’ll help me. You know, the younger generation, they are really good at it working on phones and everything like that. If I need any help with it, they’ll help.” (Patient 141)
Desired features of mHealth tobacco treatment tools	“[mHealth tools] would just help me be aware that you know, even if I want to smoke cigarettes, that they—they’re texting me and helping to get through those times, I think it would be very helpful.” (Patient 033) “Yeah, I definitely think it would be if you had like a . . . buddy system type thing, where you could sign on and have conversations with people that are going through the same thing that you’re going through. Yeah, I could see that helping.” (Patient 156) “I do kind of try to keep track of how many cigarettes I have in a day. And be aware if for some reason I’m escalating instead of slowing down.” (Patient 120) “Like if I get to the point where it’d be like, oh, I’m getting really nervous, and I don’t know whether I can do this, and well, I need to speak with a coach. A coach—somebody that’d say, hang in there, why don’t you try do this or do this or do that, or do you alternatives and everything, and somebody like that.” (Patient 159)

mHealth = mobile health.

#### Anticipated Challenges in Using mHealth Tools

Many respondents were skeptical of using an mHealth tool for tobacco treatment or smoking cessation support (Table 2). They cited personal challenges with technology as one concern, confirming the SAC’s perspective. Although many expressed discomfort and lack of regular use with mobile applications, most reported familiarity with mobile communication tools (eg, texting, email) and selected social media applications (eg, Facebook). This confirmed the SAC’s recommendation to use text messaging rather than novel app development. Respondents thought that having a tutorial video or instruction manual would be necessary for a tobacco treatment mHealth tool given their concern about the complexity of novel mHealth tools.

#### Desired Features of mHealth Tobacco Treatment Tools

Some respondents were open to the idea of using an mHealth tool for tobacco treatment support, but they identified specific desired features. For example, an option for direct interaction with another person to provide real-time user support was suggested as beneficial to facilitate initial use and engagement with a tobacco treatment mHealth tool. This reinforced the need for dedicated resources to provide a direct support hotline for all participants. Some respondents identified family members who could provide support in navigating technology if this was not available. Others suggested features included a cigarette counter, tips for avoiding smoking during cravings, having a coach or interactive support available on demand, and having an mHealth tool that could be customizable to individual needs. However, most respondents thought personal willpower was key to successful smoking cessation, and thus thought that tobacco treatment mHealth tools would only help those committed to quitting.

## Discussion

Our tobacco treatment mHealth development process included both longitudinal collaboration with a diverse group of SAC members and a qualitative study to maximize engagement of diverse, medically underserved older adults. We found that many of the adults in the target groups do have access to and use technology that could be leveraged to facilitate tobacco treatment; however, these groups require carefully designed mHealth tools and real-time technologic support to fully engage. For example, although older adults have growing access to and facility with mHealth-ready devices (eg, smartphones),^29^, ^30^, ^31^ access to a smartphone does not mean full facility with the capabilities of these technologies. Therefore, designing tobacco treatment and other mHealth tools that rely on text messaging alone would be both more inclusive (ie, accessible by those who have cell phones without smartphone technology or broadband internet access) and aligned with older medically underserved adults’ actual device use.

Additionally, although the COVID-19 pandemic increased the respondents’ mHealth and telemedicine use, barriers to uptake remain. One major barrier is the complexity of novel or unfamiliar mHealth tools. Similarly, we found that establishing a sense of personal touch is highly desired and important to patients in the design of mHealth tools. The potential for mHealth tools relies on their scalability and capacity to provide frequent points of contact, including for patients who may struggle to access other medical services.^9^^,^^32^^,^^33^ However, our findings emphasize that patients who are older and identify with groups often medically underserved desire materials and tools that provide a personal touch and emphasize they are valued and respected individuals. Culturally relevant scaffolding that includes clear introductions to the tool, personalization and customization options, real-time technology support, and a continued emphasis on the role of the individual in behavior change can provide this while supporting scalability. Such implementation efforts provide personalized attention to support uptake and use while maximizing pragmatic benefits of mHealth tools.

We provide a model for patient-centered design that incorporates community engagement through longitudinal advisors and wider representation of patient perspectives. Key insights can be derived from this approach to intervention development and refinement. The use of longitudinal advisors in the form of a community advisory board, patient and family advisory board, or SAC can facilitate iterative drafting and design of interventions and supporting materials.^34^ Our SAC represented patient perspectives and key perspectives of population health advocates, clinicians, health systems, and payors. This enabled us to balance improvements to the patient experience of intervention while maintaining a focus on pragmatism, because these simultaneous goals can conflict. The longitudinal aspect of these relationships improves bidirectional trust between advisors and investigators, reduces tokenism,^35^ and provides opportunities to iterate on design. However, gathering more diverse patient perspective than can be represented on a single committee better represents broad target populations. The semistructured interviews overcame the suggestion that patient members of the SAC must represent all patient perspectives. Thus, our model includes qualitative or mixed methods work both informed by and supplementing SAC input to enhance the utility and reliability of SAC contributions to key portions of the work.

Our study has many strengths, but also limitations. Much of our described work occurred during the COVID-19 pandemic, preventing in-person engagement during portions of the described work. In-person interviews or focus groups may have elicited broader or more nuanced patient perspectives of mHealth use. The sample of participants was diverse by design, but other important groups (ie, those with language fluency other than English and Spanish) are not represented. Although snowball sampling supplemented our standardized sampling approach, we still likely failed to reach some groups. Finally, because we incorporated the recommendations into the trial,^8^ we are unable to report on effectiveness compared with an alternative approach without such features.

## Interpretation

Because older medically underserved adults are more likely to experience tobacco-related health conditions and have less access to tobacco treatment, mHealth tobacco treatment tools should be codesigned with such groups to avoid widening such disparities.^2^^,^^9^^,^^29^^,^^36^ Longitudinal community engagement that includes broad patient perspectives facilitates the effective development and deployment of mHealth tools to maximize responsiveness to patient and community needs.

## Funding/support

This study was funded by the 10.13039/100006093Patient-Centered Outcomes Research Institute [Grant PCS-2018C1-11326].

## Financial/nonfinancialdisclosures

None declared.

## Supplementary Data

e-Online Data

e-Online Data

e-Online Data
