# Supplementary material for: Community-Engaged Development of Equitable and Scalable Mobile Health Tools for Tobacco Treatment: The Healthy Lungs Trial Experience
Source: CHEST Pulm. 2025 Jan 24;3(1):100127. doi: 10.1016/j.chpulm.2024.100127 (PMC13420469; doi:10.1016/j.chpulm.2024.100127)
Supplement: e-Online Data [file mmc2.docx]

**eAppendix 1. Interview guide**

Last Updated: March 3, 2021

*AIM: Identify the strongest barriers and most effective facilitators of delivering smoking cessation programs through mobile devices to underserved older adults*

Interviewer focus areas

- Familiarity with mobile health tools and mobile platforms in general
- Opinion of mobile health tools
- Opinions on what makes a quit tool or program successful
- Barriers and facilitators to quitting or attempting to quit

Prompt: In this interview, I'm going to ask you questions about mobile health tools. Before I ask these questions, I will explain what I mean by mobile health tools. Mobile health tools refer to: the use of mobile phones and other wireless technology in medical care. Some examples are the use of smartphone/laptop/tablet to speak with a doctor, fitness trackers like Fitbit to count steps, sending/receiving text messages to schedule appointments and connect with doctors, patient portals to access lab results, and weight loss apps like MyFitnessPal to count calories.

1. **Before we begin, are you currently smoking cigarettes at least once daily?**
   - If no, end the interview
   - If yes, proceed with the interview
2. **Please describe how comfortable you feel with the mobile health tools I just mentioned (smartphones/tablets/computers).**
   - *If which mobile devices are NOT mentioned:*
     1. What about smartphones?
     2. What about tablets?
     3. What about computers?
   - *If internet is NOT mentioned:*
     1. Do you have access to internet in your home?
        1. In your community?
   - Please describe how you use the internet on your phone or mobile device.
     1. Do you use *[insert mobile device]* for google searches?
     2. Do you use *[insert mobile device]* as a fitness tracker that counts your steps or calories?
     3. Do you use *[insert mobile device]* for social networks such as Facebook or Twitter?
     4. Do you use *[insert mobile device]* for email?
        1. Do you use email to communicate with doctors?
        2. Do you use patient portals to send messages to doctors?
     5. Do you use *[insert mobile device]* for games?
     6. Do you use *[insert mobile device]* for video calls/FaceTime?
     7. Do you use *[insert mobile device]* for text messages?
     8. Do you use your *[insert mobile device]* to watch videos?
   - What are some of the reasons why mobile health tools *might be/are* difficult for you to use?
     1. What might help with these issues?
        1. (*i.e. Paying for texting fees, vouchers for upgrading phones, mobile hot spots*)
   - Do you have someone to help you use mobile devices?
     1. If so, how do they help you?
     2. *If they don’t have someone*: how did you learn to use *[insert mobile device]?*
   - Do you remember to charge your [*insert mobile device*]?
     1. (i.e. You have a telehealth appointment but forgot to charge your phone or forgot to wear Fitbit)
3. **Can you tell me about your health care visits before the pandemic?**
   - How often did you visit your clinician? (*primary care doctor and specialists*)
   - What kind of clinician do you usually see (*doctor, nurse, nurse practitioner, PA*)?
4. Telehealth refers to the use of technology to deliver medical care. One example is using video call to speak with your doctor. Another example is talking to your nurse by phone. Many health services that usually involve in-person visits are moving onto telehealth. **Have you used any sort of telehealth services? Please describe your experience. [**If yes: continue. If no: Go to Q3a*].*
   - What did you like about the telehealth appointment?
   - What did you dislike about the telehealth appointment?
   - What instructions (if any) were made available to you?
   - Who (if anyone) helped you get started?
   - Would you be willing to use it again?
   - How did you come to use this method?
   - Had you used telehealth before the pandemic, around mid-March?
     1. If so, how has your use changed, if at all, before and after mid-March?

[If you asked Q3 go to Q4]

3a) How are you planning on getting healthcare during the pandemic?

- Have your doctor’s offices reached out to set up a phone or video call as a healthcare visit?
- Would you be willing to use a phone or video call to speak with your doctors during the pandemic?
  - Why or why not?

[We are going to change gears a bit…We are interested in learning more about the use of technology to help people quit smoking. I am going to ask you a few questions about things that might help you or others quit smoking.]

1. **Have you ever tried to quit smoking, even for just 1 day or 2?**

- If so, please describe what you tried to help you quit?
  - - (quit line, individual counseling, smoking cessation classes, medications, patches, lozenges etc.)
  - How well did they work for you?
  - How long did you quit?
  - What brought you back to smoking?
  - What do you think was missing during that quit attempt to successfully quit?
  - Would you be interested in trying to quit again?

1. **If you were thinking of quitting, what things would help you quit this time?**
   - If you don’t think you need a program, what will make you decide to quit?
     1. What would motivate you to decide to quit?
        1. Or, set a quit date?
2. **How do you think a mobile app could be used to help people quit?**
   - Have you used a quit smoking app?
     1. What was useful?
     2. What wasn’t useful/didn’t work too well?
   - What if the mobile app sent you a daily text or a reminder to quit?
     1. *If YES*: Why do you think a reminder or daily text would be helpful for you to quit?
     2. *If NO*: Why do you think a reminder or daily text wouldn’t be helpful for you to quit?
   - Would it be helpful if the mobile app allowed you to track how many cigarettes you are smoking?
     1. *If YES*: Why do you think tracking your number of cigarettes would be helpful for you to quit?
     2. *If NO*: Why do you think tracking your number of cigarettes wouldn’t be helpful for you to quit?
   - Would it be helpful if the app told you how much money you’re spend on cigarettes?
     1. *If yes*: Why do you think tracking how much money you spend on cigarettes would be helpful for you to quit?
     2. *If NO*: Why do you think tracking how much money you spend on cigarettes wouldn’t be helpful for you to quit?
3. **Would you prefer if the app allowed you to interact with real people?**
   - *If NO*: Why don’t you think interacting with real people wouldn’t be helpful for you to quit?
     1. What if you could interact with a coach or other people trying to quit in the mobile app?
   - *If yes*: What kind of people would you like to interact with in the mobile app?
     1. Coach? (to provide smoking cessation counseling)
        1. *If yes*: How would this be helpful?
        2. *If NO*: Why do you think this wouldn’t be helpful for you to quit?
     2. Other people trying to quit?
        1. *If yes*: How would this be helpful?
        2. *If NO*: Why do you think this wouldn’t be helpful for you to quit?
     3. Interact in what ways?
   - Would it be helpful to have information on how to quit?
     1. *Yes*: Why would this information be helpful for you to quit?
     2. *No*: Why would this information not be helpful for you to quit?
   - Would it be helpful to have information on the health effects of smoking?
     1. *Yes*: Why would this information be helpful for you to quit?
     2. *No*: Why would this information not be helpful for you to quit?
   - Are there any other functions in the mobile app that you think could be included in a mobile app to help people quit?
   - What do you or people you know struggle with regarding using mobile apps?
     1. Would instructions or a video be useful on how to use a mobile app?
4. **Are there any additional thoughts or comments you’d like to share with me today?**

Thank you very much for your time today. Your answers will really help our research project. Please feel free to contact us at [email] or at [phone] if you have any additional thoughts.

1. **May I please have the best mailing address to send you the $50 for your participation?**
   1. The card will arrive empty, please call us back and let us know you got the card and someone on my team will fill the card with the $50 and will be ready in 10 minutes from that for you to use.
2. **Is there a loved one in your life that** helps with: dressing, bathing, getting around the home or community or eating, taking medicine or talking to doctors and nurses, keeping you company/providing emotional support, doing shopping, managing paperwork such as bills, taking care of chores and meal preparation?
   1. Or involved in your healthcare that would like to participate?
3. **Would you like to participate in other interviews for this study? You will be sent $50 for each interview you complete.** If so, please don’t throw out the card we will be sending.
4. *If the patient mentioned wanting to quit smoking, share smoking cessation resources.*
