# Supplementary material for: Community-Engaged Development of Equitable and Scalable Mobile Health Tools for Tobacco Treatment: The Healthy Lungs Trial Experience
Source: CHEST Pulm. 2025 Jan 24;3(1):100127. doi: 10.1016/j.chpulm.2024.100127 (PMC13420469; doi:10.1016/j.chpulm.2024.100127)
Supplement: e-Online Data [file mmc3.docx]

**eAppendix 2. Codebook for interview analysis**

| Provisional Codes | Code Abbreviation | Description | Examples of Participant Responses |
| --- | --- | --- | --- |
| **In-Person Visits** | In person | Discussion of in-person visits to health care providers prior to or during the pandemic |  |
| Preference | Pref | Discussion of preference for in-person visits to health care providers |  |
| Challenges | Challenge | Challenges to in person visits to h.c. providers. Ex. Transportation, social support |  |
| **Use of Technology** |  | Use of phones, laptops, etc.  Access to internet  Ex. Patient goes to library for internet, has internet at home, uses wi-fi hotspots for internet. | Um, I'm okay with - um, like I have an iPhone, um, an Apple. So, I'm pretty comfortable with them. I don't have a tablet or a computer or anything like that, but um, I just have the regular iPhone, and I'm - I'm okay with that. |
| Assistance from others |  | Assistance in using technology from friends or family |  |
| Existing App |  | Use of mobile applications  General Apps like facebook, twitter, text, and email  Uses- discussion of how mobile apps are used by respondent |  |
| **Telemedicine** | Tele | Discussion of experience using telemedicine prior to or during the pandemic.  Any non in-person interaction with healthcare office/clinician  Ex. Calling clinician’s office to refill prescription, sending message to doctor through patient portal (myPennMedicine, Epic) |  |
| Barriers | Barr | Barriers to telemedicine Ex. Language, internet access |  |
| Benefits | Ben | Benefits of telemedicine |  |
| **Buying practices** | buy | Any mention of buying cigarettes pre-pandemic and during the pandemic.  Description of any form of cigarette acquisition.  e.g. Patient’s sister used to bring her cigarettes before the pandemic or neighbor gives patient a cigarette occasionally  e.g. Patient goes to gas station to buy cigarette pack  *N.B. cigarette buying practices either are the same or have changed due to the pandemic.* |  |
| **Prior quit attempts** | quit | Having quit; even for just 1 day or 2  Attempted to quit (even if unsuccessful). |  |
| **Smoking triggers** | trig | Mention of anything that made them want to smoke again after having quit in the past.  External factor that wants them want to smoke a cigarette after quitting or trying to quit.  *N.B. Does not include descriptions of smoking as part of daily routine.*  e.g. Mention of family member passing away, spending more time alone at home makes them want to smoke.  e.g. saw friends smoking at a party and wanted to smoke a cigarette |  |
| **Motivations to quit** | moti | Any mention of a motivation to quit now or in the past.  Any mention that makes them rethink their smoking behavior.  Any mention of wellbeing.  *N.B. Include possible/potential motivations to quit despite patient not wanting to quit.*  e.g. Mention of family, general health, etc. as incentives to quit.  e.g. Patient initiated discussion about use of a smoking cessation tool to a doctor.  e.g. Loved ones brings up their smoking and how they feel about the patient smoking. |  |
| **Smoking cessation tools/methods** | tool | Description of smoking cessation tools.  Mention of willpower needed to quit.  e.g. Doctor initiated discussion of use of smoking cessation tool.  e.g. actively trying to cut back on cigarettes  e.g. trying to buy just 1 pack at a time in order to smoke less |  |
| Barriers to smoking cessation | scba | Description of barriers to getting smoking cessation tools such as cost, no knowledge of tools, side effects of medications, etc.  Description of a tool or method not working for them  e.g. Patient has not being able to see a medical provider to talk about smoking cessation because of the pandemic  e.g. Patient describes having bad dreams after taking prescribed smoking cessation medication.  e.g. Patient says patch peels off from their skin  e.g. Misconceptions on use of smoking cessation tools (“patches injecting chemicals into veins”. |  |
| **Finances** | fina | Any description of finances related to buying cigarettes.  e.g. no impact or there is an impact on their finances |  |
| **Mental Health** | ment | Any type of psychological distress.  e.g. Cigarettes used as coping mechanism because dealing with stressful situation.  e.g. Patient expresses not being able to quit due to: cigarettes helping them face a current personal situation, cigarettes are “like a companion”  e.g. patient describes dealing with anxiety. |  |
| **Cessation app** |  |  |  |
| Features |  | Includes discussion of features that would be desirable in a smoking cessation application. |  |
